# Utilization of *Piper betle* L. Extract for Inactivating Foodborne Bacterial Biofilms on Pitted and Smooth Stainless Steel Surfaces

**DOI:** 10.4014/jmb.2212.12052

**Published:** 2023-02-28

**Authors:** Songsirin Ruengvisesh, Pattarapong Wenbap, Peetitas Damrongsaktrakul, Suchanya Santiakachai, Warisara Kasemsukwimol, Sirilak Chitvittaya, Yossakorn Painsawat, Isaratat Phung-on, Pravate Tuitemwong

**Affiliations:** 1 Department of Microbiology, Faculty of Science, King Mongkut’s University of Technology Thonburi (KMUTT), Bangkok 10140, Thailand; 2 Maintenance Technology Center, Institute for Scientific & Technological Research & Services (ISTRS), KMUTT, Bangkok 10140, Thailand; 3 Food Safety Center, ISTRS, KMUTT, Bangkok 10140, Thailand

**Keywords:** Biofilm inactivation, *Piper betle*, foodborne bacteria, pitting corrosion, stainless steel

## Abstract

Biofilms are a significant concern in the food industry. The utilization of plant-derived compounds to inactivate biofilms on food contact surfaces has not been widely reported. Also, the increasing negative perception of consumers against synthetic sanitizers has encouraged the hunt for natural compounds as alternatives. Therefore, in this study we evaluated the antimicrobial activities of ethanol extracts, acetone extracts, and essential oils (EOs) of seven culinary herbs against *Salmonella enterica* serotype Typhimurium and *Listeria innocua* using the broth microdilution assay. Among all tested extracts and EOs, the ethanol extract of *Piper betle* L. exhibited the most efficient antimicrobial activities. To evaluate the biofilm inactivation effect, *S*. Typhimurium and *L. innocua* biofilms on pitted and smooth stainless steel (SS) coupons were exposed to *P. betle* ethanol extract (12.5 mg/ml), sodium hypochlorite (NaClO; 200 ppm), hydrogen peroxide (HP; 1100 ppm), and benzalkonium chloride (BKC; 400 ppm) for 15 min. Results showed that, for the untreated controls, higher sessile cell counts were observed on pitted SS versus smooth SS coupons. Overall, biofilm inactivation efficacies of the tested sanitizers followed the trend of *P. betle* extract ≥ BKC > NaClO > HP. The surface condition of SS did not affect the biofilm inactivation effect of each tested sanitizer. The contact angle results revealed *P. betle* ethanol extract could increase the surface wettability of SS coupons. This research suggests *P. betle* extract might be utilized as an alternative sanitizer in food processing facilities.

## Introduction

Foodborne illnesses have been of paramount concern to the food industry. The US Centers for Disease Control and Prevention estimates approximately 48 million sick patients, 128,000 hospitalizations, and 3,000 deaths from foodborne illnesses per year [1]. According to the Thailand Bureau of Epidemiology, more than 100,000 annual cases of foodborne illness have been reported over the past decade [2]. Food can become contaminated at any point from farm to table as well as during manufacturing, transportation, and even in the consumer’s kitchen; consuming contaminated food will eventually result in foodborne illnesses. Ineffective cleaning and sanitizing of food contact surfaces (FCSs) in food processing plants can promote soil buildup and, in the presence of water, contribute to the formation of bacterial biofilms which may harbor pathogens [3]. Biofilms are spatially structured communities of microorganisms attached to and growing on surfaces embedded in extracellular polymeric substances (EPSs) consisting of polysaccharides, proteins, nucleic acids, and lipids [3, 4]. *Salmonella* spp. and *Listeria monocytogenes* are bacterial pathogens that can form biofilms on FCSs and have been associated with many foodborne illnesses [5-7]. Once formed on an FCS, biofilms can become more resistant to cleaning and sanitizing agents, rendering it more difficult to remove [3, 8]. It has been reported that commonly used sanitizers (*e.g.*, sodium hypochlorite, sodium hydroxide, hydrogen peroxide, and peroxyacetic acid) failed to eradicate biofilms on FCSs [9, 10]. Some commonly used sanitizing agents may also produce harmful by-products (*e.g.*, chloramine, trihalomethane, haloacetic acid, and other potentially carcinogenic compounds), cause microbial resistance [11], and corrode FCSs [12, 13].

Stainless steel (SS) is widely used in the manufacture of FCSs and equipment for food processing facilities [14]. Pitting corrosion is a common type of corrosion that usually occurs in SS due to exposure to corrosive sanitizers (*e.g.*, chlorides, bromide, and other halides) [13]. This type of corrosion is difficult to detect by visual observation because the opening is narrow, and the pits (small holes resulting from pitting corrosion) may be hidden under surface deposits. Once formed, pitting corrosion has the potential to continue to grow under SS surfaces and cause further damage inside the material [15]. In addition, pitting corrosion may promote microbial adhesion and biofilm development and could contribute to pathogen transmission from FCS to food [16].

Increasing negative perception of consumers against synthetic chemicals and sanitizers has prompted the search for natural compounds as alternatives. Plant extracts and essential oils (EOs) are mixtures of active organic compounds (*e.g.*, phenols, terpenes, aldehyde, acids, and alcohols) that can be obtained from plants and have been reported to exhibit antimicrobial and antibiofilm activities [5, 6, 11, 17-20]. However, the efficacy of plant-derived extracts or EOs to inactivate biofilms on pitted SS versus smooth has barely been reported.

Concerning the aspects above, we sought in this study to i) evaluate the antimicrobial activities of ethanol extracts, acetone extracts, and EOs obtained from seven culinary herbs (*i.e.*, *Zingiber officinale*, *Piper betle* L., *Ocimum sanctum*, *Coriandrum sativum*, *Cymbopogon citratus*, *Mentha cordifolia*, and *Ocimum basilicum*), ii) determine the biofilm inactivation effect of the selected plant extract against *S*. Typhimurium and *L. innocua* on pitted versus smooth SS samples, and iii) determine how the selected plant extract affects the wettability of the SS samples. Due to its strong genetic similarities and harmless nature, *L. innocua* has been frequently employed as a surrogate for *L. monocytogenes* [21]. Therefore, *L. innocua* was used as a surrogate for *L. monocytogenes* in this work because of laboratory biosafety regulations.

## Materials and Methods

### Preparation of Plant Materials

Culinary plants, including rhizomes of ginger (*Z. offinale*), leaves of betel vine (*P. betle*), leaves of holy basil (*O. sanctum*), seeds of coriander (*C. sativum*), aerial parts of lemongrass (*C. citratus*), leaves of spearmint (*M. cordifolia*), and leaves of sweet basil (*O. basilicum*) were purchased from a local market. The fresh samples were washed, cut into small pieces, and dried in a hot air oven (Jeio Tech, Korea) at 50°C for 24 h.

### Solvent Extraction and Acquisition of Essential Oils

After 24 h of drying, the dried plant materials (50 g) were individually submerged in 250 ml of 95% ethanol (Alcoh-A, Thailand) and acetone (Fisher Scientific, UK) at room temperature for 72 h. The extracts were then filtered through a Whatman filter paper, and the solvents were removed using a rotary vacuum evaporator (Buchi, USA) at 60°C. The extract yields (%w/w) were calculated as follows [22]:

$$\text{\%Yield=}\frac{\text{(Weight of plant extract)}}{(\text{Weight of initial sample)}}\times 100\%.$$

The EOs were kindly provided by Thai-China Flavors and Fragrances Industry Co., Ltd. The plant extracts and EOs were stored at 4°C and dissolved in 20% dimethyl sulfoxide (DMSO; Fisher Scientific) as stock solutions prior to use.

### Preparation of Microbial Cultures

S. enterica serotype Typhimurium TISTR 292 and *L. innocua* DMST 9011 were maintained on tryptic soy agar (TSA; HiMedia, India) and tryptic soy agar + 0.6% yeast extract (TSAYE; HiMedia) slants at 4°C. *S*. Typhimurium and *L. innocua* were morphologically confirmed by microscopic observation and cultivation on xylose lysine desoxycholate (XLD; HiMedia) agar and Oxford agar (HiMedia), respectively. To prepare the working cultures, loopfuls of *S*. Typhimurium and *L. innocua* were transferred to 10 ml of tryptic soy broth (TSB; HiMedia) and tryptic soy broth + 0.6% yeast extract (TSBYE; HiMedia), respectively. The cultures were incubated at 37°C for 24 h. Then, the cultures were transferred to 10 ml of TSB and TSBYE and incubated for 24 h.

### Determination of Minimum Inhibitory and Minimum Bactericidal Concentrations

The broth microdilution assay was performed to determine the minimum inhibitory concentration (MIC) and the minimum bactericidal concentration (MBC) values of plant extracts and EOs as described by Damrongsaktrakul *et al*. [23]. TSB and TSBYE were used for culturing *S*. Typhimurium and *L. innocua*, respectively. The plant extracts and EOs (100 μl) were serially 10-fold diluted in 20% DMSO in a 96-well plate to obtain initial concentrations ranging from 0.78 to 400 mg/ml. Then, 100 μl of *S*. Typhimurium and *L. innocua* in 2X TSB and TSBYE were added to the wells to obtain a concentration of approximately 5 log CFU/ml; the final concentrations of the plant extracts and EOs were 0.39 to 200 mg/ml in 10% DMSO. Negative growth controls (extracts and EOs in the test medium without bacteria) and positive growth controls (bacteria in the test medium + 10% DMSO without extracts and EOs) were also included. The plates were incubated at 37°C for 24 h. Then, 30 μl of 0.015%resazurin (Sigma Aldrich Co., USA) was added to the wells. After 30-min incubation, the plates were visually inspected. The lowest concentrations of the extracts and EOs that inhibited the growth of tested microorganisms (*i.e.*, no red or pink color) were considered the MIC. To determine the MBC values, 100 μl of the test solutions from inhibitory wells were spread on TSA plates. The plates were incubated at 37°C for 24 h. The lowest concentrations of extracts and EOs yielding ≥ 3.0 log CFU/ml reduction were deemed the MBC. The assay was completed in triplicate; each replicate was performed in duplicate (*n* = 6).

### Determination of Total Phenolic Content of *P. betle* Extract

To determine the total phenolic content of the *P. betle* ethanol extract, a Folin-Ciocalteu assay was performed as described by Damrongsaktrakul *et al*. [23] with minor modifications. The standard gallic acid solutions were prepared using 95% ethanol to obtain concentrations of 0 to 400 mg/l. The *P. betle* ethanol extract was dissolved in 95% ethanol to obtain a concentration of 500 mg/ml. Then, the standard gallic acid solutions and the *P. betle* extract were individually mixed with 0.5 ml of distilled water and 125 μl of Folin-Ciocalteu reagent, respectively. The resulting solutions were allowed to incubate for 6 min. After that, 1.25 ml of 7% sodium carbonate solution was added, followed by distilled water to bring the final volume to 3 ml. The resulting solutions were then incubated for 90 min and measured at 760 nm using a spectrophotometer (BioTek, USA) with a reference blank. The total phenolic content was represented as milligrams of gallic acid equivalent in a gram of crude extract (mg GAE/g).

### Preparation of Pitted and Smooth Stainless Steel Coupons

The type 304 SS coupons (1 cm × 2 cm) were kindly provided by AEC Industrial Services Co., Ltd. For the smooth surface condition, the SS coupons were polished using a fine grit sandpaper and rinsed with distilled water. The pitted surface condition was prepared using an electrochemical technique, according to Asaduzzaman *et al*. [24], with modification. The polished SS coupons were coated with plastic tape leaving a 1 cm × 1 cm area for electrolyte exposure. The coated SS coupons were then submerged in 50 ml of 3.5% NaCl. The DC power supply YG3005D (Yugo, Thailand) was used to provide an electrical potential (5 V) for 5 min to induce corrosion in order to generate pits on the SS coupons at room temperature. The pitted and smooth SS coupons were then cut into a size of 1 cm × 1 cm and submerged in acetone for 10 min. To sterilize them, the SS coupons were soaked in 95%ethanol for 15 min. Then, the coupons were autoclaved at 121°C for 15 min and dried in a hot air oven at 60°C for 24 h.

### Pit Measurement

Pits were observed under a Carl Zeiss Axiovert 40 MAT optical microscope (Carl Zeiss, Germany) equipped with a Dino-Eye Edge AM7025X digital camera (Dino-Lite, Taiwan), and the density of pits was expressed as numbers of pits/mm^2^. The pit diameter was determined using DinoCapture 2.0 microscope imaging software (Dino-Lite). Pit depth was determined by measuring the depth of pit cross-sections under the optical microscope using DinoCapture 2.0 microscope imaging software.

### Biofilm Formation on Stainless Steel Coupons

A cocktail inoculum of *S*. Typhimurium and *L. innocua* was prepared as described by Yegin *et al*. [25] with minor modifications. *S*. Typhimurium and *L. innocua* were centrifuged at 10,000 ×*g* at 22°C for 15 min, and the supernatants were discarded. The resulting pellets were washed in 10 ml of phosphate-buffered saline (PBS; HiMedia) and centrifuged for 15 min. Cell washing and centrifugation were completed twice. Then, *S*. Typhimurium and *L. innocua* pellets were resuspended in 0.1% peptone water (PW; HiMedia) and mixed. To form biofilm on SS coupons, the sterile coupons were placed in conical tubes containing 10 ml of TSB. Then, the cocktail inoculum was added to the tubes to achieve a target concentration of approximately 7 log CFU/ml. The tubes were incubated at 30°C for 48 h to allow biofilm formation.

### Sanitization Treatment

Following 48 h of incubation, the SS coupons were withdrawn from the conical tubes and washed three times using PBS to remove loosely attached cells. To prepare sanitizers, *P. betle* extract was dissolved in 10% DMSO to obtain the final concentration of 12.5 mg/ml. Sodium hypochlorite (NaClO; Loba Chemie, India), hydrogen peroxide (HP; QRec, New Zealand) and benzalkonium chloride (BKC, Hong Huat, Thailand) were dissolved in sterile distilled water to obtain the final concentrations of 200 ppm, 1,100 ppm, and 400 ppm, respectively. For sanitization treatment, the coupons were individually submerged in conical tubes containing 4 ml of *P. betle* ethanol extract, NaClO (pH 7), HP, and BKC for 15 min. The untreated control samples were included to assess sessile cell numbers of *S*. Typhimurium and *L. innocua* on the SS coupons without sanitization. After sanitizing treatment, the coupons were washed twice with PBS to eliminate sanitizer residues and loosely attached cells. The coupons were placed in conical tubes bearing 10 ml of 0.1% PW and sonicated at 50 Hz for 1 min (Sonics & Materials, Inc., USA). The dislodged biofilm was serially diluted in 9 ml of 0.1% PW and spread on XLD and Oxford agar plates. The resulting plates were incubated at 37°C for 24 h, and the microbial colonies were then enumerated. The limit of detection (LOD) of the assay was 0.7 log CFU/cm^2^. The assay was performed in triplicate, with two independent samples being tested per replicate (*n* = 6).

### Scanning Electron Microscopy Analysis

The untreated control and sanitizer-treated biofilm samples on pitted and smooth SS coupons were subjected to scanning electron microscopy (SEM) analysis. The coupons were washed with PBS and distilled water. The sample fixation was done by submerging the samples in 4% glutaraldehyde (Fisher Scientific) at 4°C for 2 h. Then, the coupons were washed with distilled water and gradually dehydrated in an ethanol series of 25, 50, 75, 95, and 100%, respectively. Following dehydration, samples were subjected to PdAu sputter coating and then observed under SEM (Quanta 450, FEI, USA) [23].

### Contact Angle Measurement

The contact angle measurement was performed as described by Zezzi *et al*. [26] with modifications. The pitted and smooth SS coupons were submerged in 4 ml of *P. betle* for 15 min and then washed with distilled water. To determine the effect of 10% DMSO (solvent for *P. betle* extract) on the contact angles, the SS samples were also treated with 4 ml of 10% DMSO for 15 min and then rinsed with distilled water. For the control samples, the SS coupons were immersed in distilled water for 15 min. All SS samples were then dried in a desiccator for 24 h. A sessile drop technique was used to assess the contact angle by dropping 2 μl of distilled water on the SS samples. The contact angle was measured after 10 s deposition to allow the water droplet to stabilize. An optical contact angle and interface tension meter (Model SL150, USA Kino, China) was used for the contact angle measurement.

### Statistical Analysis

Two-way analysis of variance (ANOVA) at α = 0.05 was used to determine mean differences in the populations of the tested pathogens as a function of the main effects of sanitizing treatment, surface condition of SS, and their interaction via PASW Statistics software version 17.02 (SPSS Inc., USA). The contact angle results were analyzed using one-way ANOVA. Mean separation of the pathogens and the contact angle data were performed using Fisher’s least significant difference (LSD) procedure. For the purpose of statistical analysis, 0.4 log CFU/cm^2^ was assigned to the non-detectable level of the tested pathogens.

## Results and Discussion

### Extraction Yields

The plant extraction yields are presented in Table S1. The yields from ethanol and acetone extraction ranged from 0.94 to 6.14% w/w and 0.78 to 5.84% w/w, respectively. Among all tested plant extracts, the highest yield was observed with the ethanol extract of *P. betle* (6.14% w/w). The plant parts and the type of solvents played a crucial role in the crude extract yields. Different types of organic solvents vary in polarity and capacity to permeate plant cell walls. Hence, the targeted products were distinctly dissolved and separated from the structural components [27]. Ethanol extract yields of *P. betle* have been reported in previous studies [28, 29]. Boontha *et al*. [28] reported 1.4% w/w yield, while Pin *et al*. [29] demonstrated approximately 10% yield for the ethanol extract of *P. betle*. Yield variation could be due to phytochemicals in plants, the extraction method, the extraction temperature, and the ratio of solvent to solid [29, 30].

### Minimum Inhibitory and Minimum Bactericidal Concentrations

Table 1 shows the MICs and MBCs of ethanol extracts, acetone extracts, and essential oils (EOs) of the tested plants against *S*. Typhimurium and *L. innocua*. MIC and MBC values against *S*. Typhimurium ranged from 3.125 to 200 mg/ml and 12.5 to >200 mg/ml, respectively. Overall, the acetone extract of *C. citratus* was the least inhibitory and bactericidal to *S*. Typhimurium. For *L. innocua*, MIC and MBC values varied from 3.125 to 100 and 12.5 to 200 mg/ml, respectively. For both tested microorganisms, the ethanol extract of *P. betle* displayed the lowest MICs and MBCs, and thus indicated the highest antimicrobial activity among the ethanol extracts, acetone extracts, and EOs of the tested plants. Growth of the tested pathogens was observed in the control wells, indicating that 10% DMSO showed no inhibitory effect against *S*. Typhimurium and *L. innocua*.

Hydroxychavicol, eugenol, and gallic acid have been reported to be significant phytochemical components of *P. betle* leaf, among others [31]. Hydroxychavicol has two hydroxyl groups and is more soluble in the solvent with high polarity [29]. Nguyen *et al*. [31] reported that *P. betle* leaf extracts using polar solvents showed more efficient antimicrobial activities than that with a nonpolar solvent. High contents of hydroxychavicol and eugenol were also observed with ethanol extract of *P. betle* [31]. In our study, the ethanol extract of *P. betle* exhibited higher antimicrobial activity than the acetone extract. Ethanol has higher polarity than acetone [32]. It may have dissolved hydroxychavicol and other polar or semi-polar components more efficiently than acetone, thus resulting in the *P. betle* ethanol extract having a higher amount of phytochemical components and antimicrobial activity. Previous findings have shown that *P. betle* *extract* exerted no significant adverse effects on human cells [33], rats, and mice [34-36], suggesting the safety of *P. betle* extract for human use.

### Total Phenolic Content of *P. betle* Extract

The total phenolic content of *P. betle* ethanol extract was 444 mg GAE/g. The result was within the range of the total phenolic content values reported by previous studies [27, 31, 37]. Factors affecting differences in the phenolic content across studies could be the extraction temperature, extraction method, age of plants, and conditions of the phenolic content assay.

### Pit Characteristics

**Fig. 1:**
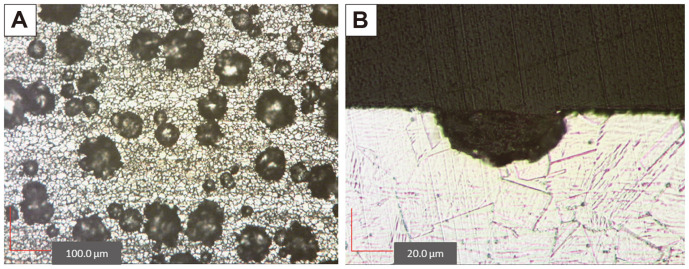
Optical microscope images of pits on stainless steel surface: (A) top view of pitted stainless steel surface, (B) cross-section of pit.

The optical microscope images of pits on SS surfaces formed by an electrochemical technique are demonstrated in Fig. 1. According to ASTM G46-94 (2018) [38], pits in this study appeared to have a “wide, shallow” shape (Fig. 1B). It should be noted that the pit formation was conducted for 5 min; therefore, longer exposure time to an electrical potential may have resulted in changing the shape of pits. The average size of simulated pit mouths was 63.3 ± 27.3 μm in diameter. Several aspects, such as the inhomogeneity of the SS alloys during manufacture, could cause a broad range of pit sizes. This could result in different resistance to corrosion locally. Therefore, the pit size, which was the result of pitting corrosion, could be varied accordingly. The depth of the simulated pits was 25.7 ± 10.6 μm. The same reason was applied to the variation of the pit depth. In addition, the method for determining pit depth could also affect the measurement results, such as the selection of the pit for evaluation and the pit cross-sectioning method used. Another aspect of determining the extent of the pitting is the pit density, which in this case was 41.6 ± 9.2 pits/mm^2^.

### Biofilm Inactivation Effect of Tested Sanitizers

**Fig. 2:**
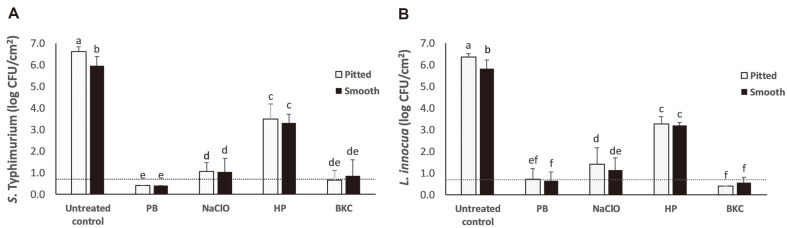
Means of surviving *S*. Typhimurium (A) and *L. innocua* (B) obtained from the mixed-culture biofilms on the pitted and smooth stainless steel coupons following sanitization treatment. The sanitizers were 12.5 mg/ml *P. betle* extract (PB), 200 ppm sodium hypochlorite (NaClO; pH 7), 1100 ppm hydrogen peroxide (HP), and 400 ppm benzalkonium chloride (BKC). Means sharing a different letter are significantly different (*p* < 0.05). Bars represent means; error bars represent standard deviations of three independent replicates. The horizontal dotted line depicts the limit of detection of the assay (0.7 CFU/cm^2^).

According to the MIC and MBC results, the ethanol extract of *P. betle* was selected for the biofilm inactivation assay compared with the commonly used sanitizers, *i.e.*, NaClO, HP, and BKC. Fig. 2 shows the means of surviving *S*. Typhimurium and *L. innocua* obtained from the mixed-culture biofilms on the pitted and smooth SS coupons following sanitization treatment. For *S*. Typhimurium, the sessile cell population on the untreated, pitted SS control (6.6 ± 0.2 log CFU/cm^2^) was significantly higher (*p* < 0.05) than those on the untreated, smooth SS control (6.0 ± 0.4 log CFU/cm^2^) (Fig. 2A). After sanitization treatment, the *S*. Typhimurium cell numbers were reduced to the range of below detectable level to 3.5 ± 0.7 log CFU/cm^2^. For *L. innocua*, a significant difference (*p* <0.05) was also observed between the sessile cell numbers on the untreated, pitted SS control (6.4 ± 0.2 log CFU/cm^2^) and the population on the untreated, smooth SS control (5.8 ± 0.4 log CFU/cm^2^) (Fig. 2B). The sessile cell numbers of *L. innocua* ranged from below detectable level to 3.5 ± 0.3 log CFU/cm^2^ after treatment with the tested sanitizers. For each tested pathogen, the sessile cell numbers did not differ when treated with the same sanitizer (*p* > 0.05) regardless of the SS surface conditions. Our preliminary data showed that 10% DMSO had no significant inactivation effect on biofilms of *S*. Typhimurium (Table S2) and *L. innocua* (Table S3).

Overall, the biofilm inactivation effect of the tested sanitizers followed the pattern *P. betle* extract ³ BKC > NaClO > HP. *Piper betle* L. is a medicinal plant that belongs to the Piperaceae family with an origin in Southeast Asia [39]. It has been reported to possess anticarcinogenic, antimutagenic, anti-inflammatory, antibiofilm, and antimicrobial activities [31, 40]. However, the study on the biofilm inactivation efficiency of *P. betle* extract on FCSs is still lacking. In this study, *P. betle* extract exhibited the most significant biofilm inactivation effect among the tested sanitizers. The qualitative phytochemical analysis by Syahidah *et al*. [41] demonstrated the presence of phenols, flavonoids, alkaloids, tannins, saponins, glycosides, terpenoids, and steroids in the *P. betle* leaf extract. Hydroxychavicol and eugenol are the major phenolic compounds in *P. betle* leaves [31, 42]. Hydroxychavicol could induce DNA damage and suppress cell division, resulting in bacterial cell death [43]. For eugenol, the hydroxyl group could interact with microbial proteins and disrupt the enzyme function, leading to non-specific membrane permeability and inhibition of ions and ATP transport [31]. NaClO, HP, and BKC are commonly used sanitizers in food processing facilities [44]. NaClO has strong oxidizing activity and can oxidize the sulfydryl groups on certain enzymes in the cell membrane or protoplasts, resulting in impairment of cellular protein activities. Also, it may induce irreversible decarboxylation reactions [45]. HP is a strong oxidant and has been reported to damage bacterial DNA, proteins, and cellular membranes [46]. BKC is a quaternary ammonium compound; it can perturb and disrupt the membrane bilayers by the alkyl chains and disrupt the charge distribution of the microbial membrane by the charged nitrogen [47]. Robbins *et al*. [46] demonstrated 100 ppm chlorine decreased the biofilm of *L. monocytogenes* on stainless steel chip by 6.49 log CFU/chip after 10 min of exposure. Damrongsaktrakul *et al*. [23] showed 3.37 log CFU/cm^2^ reduction of *S*. Typhimurium on SS samples after 30 min treatment with NaClO. The study by Kostaki *et al*. [48] revealed that 6 min exposure to 50 ppm BKC produced < 4 log CFU/cm^2^ and < 3 log CFU/cm^2^ reduction of the biofilms of S. enterica and *L. monocytogenes* (monospecies and dual species), respectively. In our study, NaClO and BKC reduced the biofilms of *S*. Typhimurium and *L. innocua* on pitted and smooth SS samples by > 5 log CFU/cm^2^. Nevertheless, NaClO failed to decrease biofilms of the tested pathogens to below the detectable level, while BKC was able to do so for *L. innocua* biofilms. The variation in sanitizer efficacy across studies might be attributable to the concentration of sanitizers, contact time, temperature, biofilm age, growth condition, and microbial type. For HP, reduction of < 4 log CFU/cm^2^ of the tested pathogens was obtained in our study. *S*. Typhimurium and *L. innocua* are catalase-positive microorganisms [49, 50]. Therefore, HP might be degraded by the tested pathogens, making it less efficient to inactivate biofilms than other tested sanitizers.

Pits and crevices could facilitate bacterial adhesion and biofilm formation on SS surfaces [9, 16]. In the present study, the sessile cell numbers in the biofilms of *S*. Typhimurium and *L. innocua* on pitted SS were significantly greater than those on smooth SS samples. However, the sessile cell numbers of each tested pathogen on smooth versus pitted SS surfaces were not significantly different when treated with the same sanitizer. This may have been due to the large pit size on SS surfaces, which allowed sanitizer access to the pits. In agreement with our study, Kim *et al*. [51] reported higher counts of *Staphylococcus aureus* biofilm on scratched SS surfaces vs. smooth SS surfaces. No significant difference in chlorine (200 ppm) efficacy against biofilms on smooth and scratched SS surfaces was observed. In another study, the efficacy of 200 ppm chlorine dioxide, 70% alcohol, and 200 ppm quaternary ammonium compound against the biofilm of *Bacillus cereus* did not differ on smooth versus scratched SS surfaces after 10 min sanitization [52]. However, the sizes of scratches or crevices were not reported in these studies.

### Scanning Electron Microscope Imaging

**Fig. 3:**
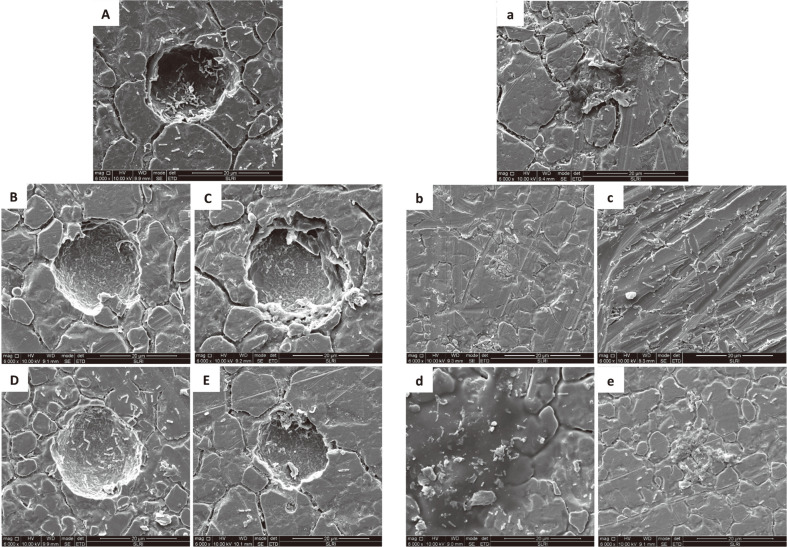
Scanning electron microscope images of the mixed culture of *S*. Typhimurium and *L. innocua* biofilms on pitted (uppercase letters) and smooth (lowercase letters) stainless steel coupons after sanitization treatment. Untreated control (A,a); *Piper betle* extract (B,b); NaClO (C,c); HP (D,d); BKC (E,e).

The SEM images of the mixed culture of *S*. Typhimurium and *L. innocua* biofilms on pitted and smooth stainless steel coupons after treatment with sanitizers are represented in Fig. 3. The untreated control samples on both SS surface conditions (Figs. 3A, 3a) showed intact, rod-shaped cell morphology with EPS. In addition, the sessile cells of the mixed culture were also observed in the pit of the SS coupon. The samples treated with *P. betle* extract (Figs. 3B, 3b) and BKC (Figs. 3E, 3e) showed a great reduction in the sessile cell numbers on both SS surface conditions. However, less reduction in the sessile cell numbers was observed with samples exposed to NaClO (Figs. 3C, 3c) and HP (Figs. 3D, 3d). A thick EPS layer was still present in the sample treated with HP (Fig. 3d).

In agreement with the plate count results, the SEM images showed greater amounts of sessile cells of the mixed-culture biofilms on pitted versus smooth SS coupons. This indicated pits/crevices could allow for microbial attachment and biofilm formation. In line with our findings, the SEM images by Kim *et al*. [51] revealed more sessile cells of *S. aureus* biofilm on scratched SS surfaces compared to smooth surfaces. In our study, *P. betle* extract showed the greatest biofilm removal on both SS surface conditions, suggesting that *P. betle* extract may be employed as an alternative to the commonly used sanitizers in food processing facilities. For other tested sanitizers, lower numbers of sessile cells were observed after sanitization, indicating that these sanitizers could partially remove biofilms of *S*. Typhimurium and *L. innocua*. In addition to inactivating microorganisms, chlorine is known to remove EPS from surfaces [53, 54]. Agreeing with our study, SEM images from previous research showed that commonly used sanitizers failed to completely eradicate microbial biofilms on SS surfaces [23, 55]. It is worth noting that our study employed the submersion method for sanitization. Therefore, the sanitization efficacy may be improved by the combination of chemical, biological, and physical treatment [56].

### Contact Angles of Stainless Steel Samples

To determine how *P. betle* extract affected the wettability of the SS samples, the pitted and smooth SS samples treated with *P. betle* extract were subjected to contact angle measurements (Table 2). For either surface condition, 10% DMSO showed no significant effect (*p* > 0.05) on the contact angle values of the SS coupons versus controls. The contact angle values of the SS samples treated with *P. betle* extract were significantly lower (*p* < 0.05) than those of the controls. Results indicated that *P. betle* extract could increase the hydrophilicity of the SS samples; this could be due to the hydroxyl group and the polar bioactive compounds in *P. betle* extract. Greater hydrophilicity allows a sanitizer to spread on the surface more easily (greater wettability) [57]. When applied to biofilms, it could be deduced that *P. betle* extract may have increased the wettability of the biofilms, resulting in more efficient sanitization.

## Conclusion

Overall in this study, the biofilm inactivation efficacy of the tested sanitizing agents against *S*. Typhimurium and *L. innocua* biofilms on pitted and smooth SS surfaces followed the trend of *P. betle* extract ³ BKC > NaClO > HP. The surface conditions of SS samples did not affect the biofilm inactivation efficacy of all tested sanitizers. *P. betle* extract increased the wettability of the pitted and smooth SS samples. This study indicates that *P. betle* extract may be an alternative to frequently used sanitizers for removing biofilms from food contact surfaces in food processing environments.

## Supplemental Materials

Supplementary data for this paper are available on-line only at http://jmb.or.kr.

## Figures and Tables

**Table 1:** Minimum inhibitory concentrations (MICs) and minimum bactericidal concentrations (MBCs) of ethanol extracts, acetone extracts, and essential oils against *S*. Typhimurium and *L. innocua*.

	*S*. Typhimurium						*L. innocua*					
	
	MIC (mg/ml)			MBC (mg/ml)			MIC (mg/ml)			MBC (mg/ml)		
			
Plant Species	EtOH	Ace	EO	EtOH	Ace	EO	EtOH	Ace	EO	EtOH	Ace	EO
*Z. officinale*	25	50	25	100	200	50	50	50	25	100	100	100
*P. betle*	3.125	12.5	12.5	12.5	25	25	3.125	12.5	25	12.5	50	50
*O. sanctum*	100	100	25	200	200	50	50	100	12.5	100	200	50
*C. sativum*	100	50	12.5	200	200	50	100	50	12.5	100	100	50
*C. citratus*	50	200	50	200	>200	25	50	100	25	200	200	50
*M. cordifolia*	100	100	25	100	200	50	100	50	50	200	200	100
*O. basilicum*	25	50	25	100	100	50	12.5	25	100	25	50	200

Ethanol (EtOH); Acetone (Ace); Essential oil (EO)

**Table 2:** Contact angle measurements of pitted and smooth stainless steel coupons treated with DMSO and *P. betle* extract.

Treatment	Contact angle (°)
Control (pitted SS)	101.39 ± 5.05^ab^
Control (smooth SS)	94.15 ± 6.68^c^
DMSO (pitted SS)	104.28 ± 2.06^a^
DMSO (smooth SS)	96.15 ± 2.69^bc^
*P. betle* (pitted SS)	17.79 ± 2.54^d^
*P. betle* (smooth SS)	16.27 ± 4.42^d^

Means sharing a different letter are significantly different (*p* < 0.05).
