# Supplementary material for: Utilization of Piper betle L. Extract for Inactivating Foodborne Bacterial Biofilms on Pitted and Smooth Stainless Steel Surfaces
Source: J Microbiol Biotechnol. 2023 Feb 28;33(6):771–9. doi: 10.4014/jmb.2212.12052 (PMC10331948; doi:10.4014/jmb.2212.12052)
Supplement: Supplementary file 1 [file jmb-33-6-771-supple.pdf]

J. Microbiol. Biotechnol.
https://doi.org/10.4014/jmb.2212.12052

Supplementary Tables

Table S1. Yield (%w/w) of plant extracts from ethanol and acetone extraction.

Plant species	Common name	Plant material	Ethanol extract yield (%w/w)	Acetone extract yield (%w/w)
Zingiber offinale	Ginger	Rhizomes	1.42	1.21
Piper betel	Betel vine	Leaves	6.14	5.84
Ocimum sanctum	Holy basil	Leaves	2.67	2.29
Coriandrum sativum	Coriander	Seeds	0.94	0.78
Cymbopogon citratus	Lemongrass	Aerial parts	5.23	3.41
Mentha cordifolia	Spearmint	Leaves	2.41	2.33
Ocimum basilicum	Sweet basil	Leaves	2.91	2.88

Table S2. Mean survivors (log CFU/cm^2) of *S.* Typhimurium sessile cells obtained from the mixed culture biofilms on stainless steel coupons after treatment with 10% dimethyl sulfoxide.

Treatment	Mean survivors (log CFU/cm^2)
Control (pitted SS)	6.6 ± 0.5[a]
DMSO (pitted SS)	6.4 ± 0.8[ab]
Control (smooth SS)	5.7 ± 0.3[bc]
DMSO (smooth SS)	5.6 ± 0.7[c]

Means sharing a different letter are significantly different ($p < 0.05$).

Table S3. Mean survivors (log CFU/cm^2) of *L. innocua* sessile cells obtained from the mixed culture biofilms on stainless steel coupons after treatment with 10% dimethyl sulfoxide.

Treatment	Mean survivors (log CFU/cm^2)
Control (pitted SS)	6.7 ± 0.7^{a}
DMSO (pitted SS)	6.5 ± 1.0^{ab}
Control (smooth SS)	5.7 ± 0.4^{bc}
DMSO (smooth SS)	5.4 ± 0.5^{c}

Means sharing a different letter are significantly different ($p < 0.05$).
